# Solution Structure of the 2A Protease from a Common Cold Agent, Human Rhinovirus C2, Strain W12

**DOI:** 10.1371/journal.pone.0097198

**Published:** 2014-06-17

**Authors:** Woonghee Lee, Kelly E. Watters, Andrew T. Troupis, Nichole M. Reinen, Fabian P. Suchy, Kylie L. Moyer, Ronnie O. Frederick, Marco Tonelli, David J. Aceti, Ann C. Palmenberg, John L. Markley

**Affiliations:** 1 National Magnetic Resonance Facility at Madison, Biochemistry Department, University of Wisconsin-Madison, Madison, Wisconsin, United States of America; 2 Center for Eukaryotic Structural Genomics, Biochemistry Department, University of Wisconsin-Madison, Madison, Wisconsin, United States of America; 3 Institute for Molecular Virology, University of Wisconsin-Madison, Madison, Wisconsin, United States of America; Centro Nacional de Biotecnologia – CSIC, Spain

## Abstract

Human rhinovirus strains differ greatly in their virulence, and this has been correlated with the differing substrate specificity of the respective 2A protease (2A^pro^). Rhinoviruses use their 2A^pro^ to cleave a spectrum of cellular proteins important to virus replication and anti-host activities. These enzymes share a chymotrypsin-like fold stabilized by a tetra-coordinated zinc ion. The catalytic triad consists of conserved Cys (C105), His (H34), and Asp (D18) residues. We used a semi-automated NMR protocol developed at NMRFAM to determine the solution structure of 2A^pro^ (C_105_A variant) from an isolate of the clinically important rhinovirus C species (RV-C). The backbone of C2 2Apro superimposed closely (1.41–1.81 Å rmsd) with those of orthologs from RV-A2, coxsackie B4 (CB4), and enterovirus 71 (EV71) having sequence identities between 40% and 60%. Comparison of the structures suggest that the differential functional properties of C2 2A^pro^ stem from its unique surface charge, high proportion of surface aromatics, and sequence surrounding the di-tyrosine flap.

## Introduction

**Figure 1:**
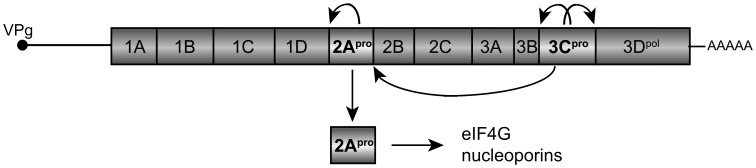
An RV RNA genome encodes a single polyprotein. The polyprotein is cleaved co- and post-translationally to release mature viral proteins. During infection, 2A^pro^ is excised at the N-terminus by self-catalysis and at the C-terminus by 3C^pro^. The released protease cleaves cellular substrates including eIF4G and nucleoporins.

Human rhinoviruses (RVs) are single-stranded, positive-sense RNA *Enteroviruses* in the *Picornaviridae* family and the most ubiquitous agents of the common cold. Originally catalogued by serotyping relative to an historical repository of clinical strains, thousands of isolates representing more than 110 different RV genotypes are now binned within the RV-A and RV-B species, according to overt similarities in their VP1 capsid sequences. For taxonomic clarity, the species letter (e.g. A or B) precedes the assigned type number (e.g. B14, A2) when referring to individual clades. Like other enterovirus genomes, the RVs encode a polyprotein that is co- and post-translationally processed by proteases that form part of the polyprotein (Figure 1). The first cleavage is by 2A^pro^. It occurs autocatalytically within the nascent polyprotein to form the amino terminus of the protease. The downstream 3C^pro^ subsequently undergoes two self-release reactions and then completes the excision of 2A^pro^.

During infection, both enzymes contribute to host cell shut-off activities, helping the virus evade host defense mechanisms and promote its replication. Among known reactions, 3C^pro^ and/or its precursors cleave nuclear transcription factors, preventing most pol2 mRNA synthesis [1], [2]. In parallel, 2A^pro^ targets translation pathways by cleaving initiation factors eIF4G-I and -II, required proteins for cap-dependent mRNA recognition by ribosomes [3], [4]. Additionally, 2A^pro^ reacts with the nuclear pore complex, cleaving multiple central core nucleoporin proteins (Nups). Since the movement of cellular proteins and RNA in and out of the nucleus is at the core of all gene activation schemes, including those required for nearly every innate immunity trigger, the 2A^pro^ alteration of Nups results in a comprehensive failure of nucleocytoplasmic transport and dependent processes of intracellular signaling [5], [6]. Interestingly though, few of the homologous enterovirus 2A^pro^ behave exactly the same with regard to these activities [7]. Among RV genotypes, the pairwise 2A^pro^ sequence identities range from 33% to 98% [8], a variation much greater than for the respective 3C^pro^ (<20%), or even some regions of the capsid proteins [8]. The variation confers to each 2A^pro^ subtle differences in substrate preference and rate kinetics toward particular Nups and eIF4G cohorts [9]. The observed turnover rates varied in the order: HRV-A > HRV-C >> HRV-B. The individual proclivities are not well understood, but they are proposed to be linked mechanistically to diverse infection outcomes unique to each sequence clade, perhaps through the regulation of preferential cytokine induction [9].

The enterovirus 2A^pro^ are small (142–150 amino acids) chymotrypsin-like enzymes that use Cys as the active nucleophile [10], [11]. The crystal structures of RV-A2 [11] and EV-71 (enterovirus 71) [12], [13] and the NMR structure of EV-CB4 (enterovirus coxsackie B4) [14] enzymes have been determined. When combined with biochemical studies on RV-B14, the structures show these enzymes are able to choose their preferred substrates from among a variety of related sequences because their highly variable binding surfaces sense and discriminate residues P8 to P2′ relative to the scission position [15]. The discernment influences the cleavage rates and pattern selection of many cellular substrates as well as the precise location of the polyprotein self-processing sites [16], [17]. From an antiviral standpoint, it is important to understand how this selectivity works at the structural level for different 2A^pro^, because putative therapies aimed at the plethora of RV types need to define and target commonalities among the crucial viral enzymes.

In 2006, multiple rhinoviruses representing a new species, the RV-C, were discovered in patients suffering influenza-illnesses with severe respiratory compromise [18]. The RV-C have special clinical relevance, because it is now recognized these new isolates (51 types) can grow in both the upper and lower airways and are responsible for up to half of RV infections in children, especially those with a propensity for asthma. Unlike the RV-A or RV-B, the RV-C cannot be grown in established tissue culture, a limitation that has hindered investigations into interventions directed against the virus capsid, or viral enzymes. Nonetheless, multiple RV-C genomes have been sequenced in their entirety, and key isolates have been rendered into cDNA [19]. These reagents have allowed essential non-structural proteins to be expressed and compared at the enzymatic level, including the 2A^pro^ from types C2 and C15 [9]. We report here the first 3D structure of an RV-C protein, the 2A^pro^ from C2, strain W12, whose functional properties have been studied extensively [9]. Stable isotope-labeled protein was prepared at the Center for Eukaryotic Structural Genomics (CESG), and the solution structure was determined at the National Magnetic Resonance Facility at Madison (NMRFAM). In addition to achieving the goal of providing biological insights into the intrinsic enzyme variability, the full, extensive NMR data collected served as test sets for NMRFAM software designed for high-throughput structure determination, including PINE-SPARKY [20] and PONDEROSA [21].

## Materials and Methods

### Plasmid Design and Construction

The protease cDNA was from RV-C2, strain W12 [9]. The sequence of the 2A gene was identical to GenBank JN837695, although the parental genome has not been sequenced entirely [22]. An amplicon for the gene encoding the RV-C2 2A^pro^ (strain W12) was isolated by PCR methods from the pET-11a plasmid previously described as Cw12 [9]. The reaction used AccuPrime Supermix (Invitrogen) and DNA primers 5' 2A^pro^-Bsa1 and 3' 2A^pro^-Xho1 (UW-Madison Biotechnology Center) shown in Table 1. The PCR product and DNA for expression vector, pE-SUMO Kan (Lifesensors) were digested with BsaI (New England Biolabs) and XhoI (Promega) then ligated by T4 DNA ligase under a temperature cycling reaction at 10°C for 30 s and 30°C for 30 s, repeated 800 times. Competent *E. coli* cells (Lucigen 10G) were transformed with a heat-inactivated ligation sample (65°C for 25 min) then plated onto YT agar plates containing kanamycin (50 µg/mL). After overnight incubation (37°C), individual colonies were picked, suspended and stored in 20% sterile glycerol. The cell suspensions (3 μL glycerol stocks) were screened by PCR and positive recombinant plasmids were isolated and the inserted DNA was sequenced (UW-Madison Biotechnology Center) to identify clones with intact 2A^pro^ genes. Site-directed mutagenesis to convert the active site-Cys_105_ codon to Ala_150_ used primers PI 5' 2A^pro^-C_105_A and PI 3' 2A^pro^-C_105_A (Table 1), with polymerase incomplete primer extension (PIPE) methods and either AccuPrime Supermix or Stratagene Pfu Turbo Ultra [23]. In preliminary extraction trials, this modification (pC2-2A-C_105_A) gave larger, more stable yields of 2A^pro^ for structure studies.

**Table 1:** DNA Primers used for Cloning and Mutating RV-C2 2A^pro^.

	DNA primer name	Primer DNA sequences*
1	5' 2A^pro^-Bsa1	5′ACTAGTGGTACCG**GTCTCA**AGGT GGACCTAGTGACCTATTTGTTCAC
2	3' 2A^pro^-Xho1	5′GGGCCCG**CTCGAG**GGATCCTCATTA TTGAGAGGTTGCTTTGATATTATAAG
3	PI 5' 2A^pro^-C_105_A	CCA GGT GAC gcg GGA GGT AAA TTA CTG TGC AGA CAT GGG GTT
4	PI 3' 2A^pro^-C_105_A	TTT ACC TCC cgc GTC ACC TGG GAC ACA TGG TCC TTC TCC AAT

*Restriction sites are in bold; primer regions that anneal to 2A^pro^ gene are underlined; and lowercase letters show DNA bases at the sites of directed mutagenesis.

### Optimal Expression Parameters

Host selection for optimal 2A^pro^ production used small-scale screening techniques developed by the CESG [24]. A series of competent *E. coli* strains (Rosetta2(DE3), Rosetta2(DE3)-pLysS from Novagen, and BL21-DE3 CodonPlus RILP from Stratagene) were transformed with pE-SUMO C2 2A^pro^ then grown on plates containing chloramphenicol and kanamycin (either YT agar plus 1% glucose or MDAG solid medium). The plates were incubated (37°C) overnight, before colonies were picked into MDAG liquid medium [25] (0.5 mL, supplemented with the appropriate antibiotics) in a 96-well format growth block. The composition of MDAG solid medium and MDAG liquid medium can be found in Protocol ID: LP.4813 at http://sbkb.org/tt/protocol?ttid=MPP-GO.111408&lab=MPP&trialid=3&protocolid=LP.4813.

The cultures were grown overnight a 25°C with shaking at 250 rpm. 10–20 μL of each culture was used to inoculate 0.5 mL of Terrific Broth with glycerol (TB+g) auto-induction medium prepared in a series of 96-well format growth blocks. The blocks were shaken and incubated at varying temperatures (30, 25, 15 and 10°C) to identify the best combinations of host strain, growth temperature and induction methods for soluble protein overproduction, as assayed by SDS-PAGE analysis of the soluble fractions and spin IMAC (immobilized metal affinity chromatography) captured protein.

### Large-Scale Protein Production

For large-scale production of 2A^pro^, cell cultures were amplified from fresh transformations of BL21(DE3) with the pE-SUMO C2 2A^pro^ plasmid. Colonies were inoculated into starter cultures (1 mL YT, plus 1% glucose, kanamycin and chloramphenicol). After initial growth with shaking (1 to 3 h, 37°C, 250–320 rpm), the starters were transferred into MDAG (50–100 mL plus antibiotics) then further grown overnight (25°C, rotary shaker, 250–320 rpm). These starter cultures (10–12 mL) were then amplified in 2 L PET bottles (500 mL YT medium in a rotary shaker) for 2–5 h, until the OD_600_ was between 1.0 and 1.4 AU. Growth temperature was reduced to 25–30°C, ZnCl_2_ was added (to 50 µM), followed 15–30 min later by IPTG (to 0.1–0.2 mM). The cells were grown overnight with shaking (250–320 rpm), harvested by centrifugation (4,000 g, 30 min) and stored at −80°C. In tests to optimize protein yields, unlabeled 2A^pro^ was also prepared using 500 mL of TB+g based auto-induction medium [26]. Essentially, this is a basic medium (12 g/L tryptone, 24 g/L yeast extract, 9.4 g/L KH_2_PO_4_, 2.2 g/L K_2_HP O_4_ and 10 g glycerol, and 100 μL/L antifoam) with supplements (3.75% aspartic acid, 2 mM MgS O_4_, 0.825 mM glucose, 87 mM glycerol, 4.6 mM α–lactose). The TB+g auto-induction medium was used in place of YT and required no induction with IPTG.

### Preparation of Uniformly ^15^N and ^13^C/^15^N-Labeled Protein on a Large-Scale

Isotopically-labeled protein was prepared as described above, except that an M9 based medium was used in place of YT (per L: 100 mL of 10x M9 salts, 70 g Na_2_HPO_4_, 30 g KH_2_PO_4_, 5 g NaCl, 1 mL of 1000x metal mix, 1 mL of B12 vitamin mixture [25], [26], 30 mg thiamine, 100 μL antifoam, 35 µg/mL chloramphenicol and 50 µg/mL kanamycin [26] and, as appropriate, 1 g ^15^NH_4_Cl and/or 4 g U-^13^C-glucose). The medium also contained 0.1 mM CaCl_2_, 50 µM ZnCl_2_, and 2 mM Mg_2_SO_4_.

### Protein Purification

**Figure 2:**
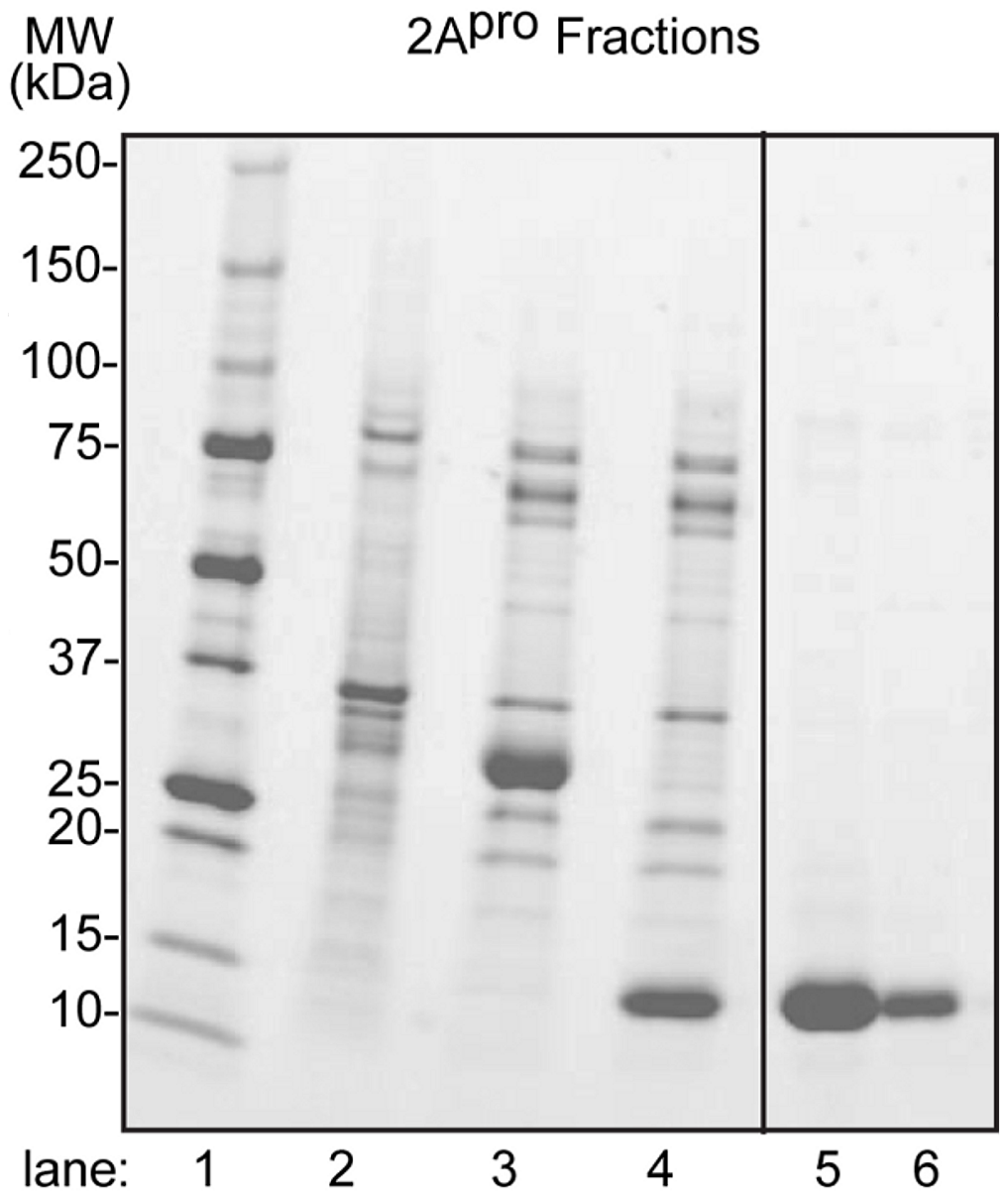
SDS-PAGE illustrating purification of RV-C2 2A^pro^. The recombinant methods described above were used to prepare ^13^C/^15^N-labeled C2 2A^pro^ (C_105_A) for NMR studies. Representative samples from the procedure were fractionated by SDS-PAGE then visualized with Bio-Rad Stain-Free. Lane 1, Bio-Rad Precision Plus protein standards; lane 2, protein pellet after (NH_4_)_2_SO_4_ precipitation; lane 3, SUMO-2A^pro^ after IMAC elution; lane 4, 2Apro after SUMO cleavage and IMAC elution; lanes 5–6, final protein fractions after gel filtration.

Cell pastes (5–10 g) were thawed and resuspended in lysis buffer (60–70 mL, 20 mM Tris pH 7.2, 500 mM NaCl, 10% ethylene glycol, 5 mM imidazole, 1 mM PMSF, 0.1% NP-40, Sigma) containing lysozyme (5 μL, Novagen), RNase (10 μL, Qiagen), Benzonase (5 μL, Novagen, 25 U/µl), or OmniCleave nuclease (Epicenter, 10 KU). The lysates were sonicated in a Misonix 3000 at 4°C with pulsing on (∼80 Watt) for 2 s and off for 4 s over 15 min and then clarified by centrifugation (30 min, 70,000 g). Polyethylene imine (to 0.1% w/v, Fluka) was added, and the samples were clarified again by centrifugation (30 min, 70,000 g) before the addition of (NH_4_)_2_SO_4_ (to 70% w/v) and DTT (to 2 mM). The collected pellets were resuspended in IMAC buffer 1 (30–40 mL, 20 mM Tris, pH 7.2, 10% glycerol, 35 mM imidazole, 1 mM PMSF), clarified (70,000 g, 30 min) then filtered (0.8 micron, Millipore) before loading onto IMAC resin (Qiagen Superflow FF) at a rate of 1–2 mL/min. The column (∼10 mL) was washed (10 volumes) with IMAC buffer 2 (buffer 1 plus 500 mM NaCl) then with IMAC buffer 3 (buffer 2 plus 65 mM imidazole), before protein elution with IMAC buffer 4 (buffer 2 plus 250 mM imidazole). Usually, 90% of the target was eluted in the first 15–30 mL as assayed by SDS-PAGE. Appropriate fractions were dialyzed overnight into buffer (Tris 20 mM pH 8.0, 150 mM NaCl and 2 mM DTT or β-mercaptoethanol), before the SUMO domain was removed from the N-terminus of 2A^pro^ by incubation with 0.5 mg SUMO protease (prepared in house) for 3–4 h at 30°C. The sample was loaded onto an IMAC column freshly equilibrated with IMAC buffer 1, which bound the His-tagged SUMO domain. The 2A^pro^ target was retrieved in the flow-through (4–5 fractions of 5–10 mL) and pooled. The final fractionation was by gel filtration (GE Healthcare HiPrep 16/60 Sephacryl S-200, 20 mM Tris, pH 8.0, 150 mM NaCl, 2 mM DTT). The purified protein was spin concentrated (Sartorius Vivaspin 20 10 kDa PES concentrator, 5,000 g) and then drop frozen in liquid nitrogen. The final yield was 27.5 mg of purified protein from 0.5 L double-labeled Martek (rich) media. The purity of protein samples was determined by SDS-PAGE (Figure 2). The C_105_A variant protein aggregated less during purification and produced a higher yield of protein.

#### NMR Data Collection

The samples for NMR spectroscopy contained 3.4 mg [U-^13^C,U-^15^N]-2A^pro^ dissolved in buffer (0.4 mL, 10 mM MES, 20 mM NaCl, 10 mM DTT, 10% ^2^H_2_O, 90% H_2_O, pH 6.5). The solutions (∼0.5 mM) were placed in 5 mm Shigemi tubes (Allison Park, PA). NMR data were collected at NMRFAM on Agilent VNMRS spectrometers operating at 600 MHz, 800 MHz, and 900 MHz. The temperature was regulated at 313 K, the temperature at which the protein exhibited the best quality 2D ^1^H-^15^N HSQC spectrum. A 600 MHz spectrometer equipped with a triple-resonance cryogenic probe was used to record 3D HNCO, HN(CA)CO, HNCA, HN(CO)CA, CBCA(CO)NH, HBHA(CO)NH, C(CO)NH, H(CCO)NH, H(C)CH-TOCSY, and ^15^N-edited NOESY data sets. The 800 MHz spectrometer with a conventional triple-resonance probe was used to record 2D ^1^H-^15^N HSQC, 3D ^15^N-edited TOCSY, (H)CCH-TOCSY, and ^13^C-edited NOESY data sets. The 900 MHz instrument with a triple-resonance cryogenic probe was used to record 2D ^1^H-^13^C HSQC and 3D HNCACB spectra. All time-domain data were processed with NMRPipe [27] to generate frequency-domain sets which were converted to SPARKY (ucsf) file format [28] for further analysis.

#### NMR Spectral Analysis and Structure Calculation

Resonances for backbone atoms in the ^1^H-^15^N HSQC, HNCACB, and CBCA(CO)NH spectra were initially identified with the APES program [29]. The restricted peak picking feature in SPARKY identified signals from additional backbone and side chain atoms. All peaks identified by automation were carefully validated by visual inspection. Peak lists for each spectrum were exported to the PINE-NMR server [30], which yielded automated resonance assignments for all but four of the backbone spin systems. The assignment probabilities were high for all but one residue, which was at 50%. We used the PINE-SPARKY [20] package to validate these assignments and complete the missing assignments. Validated chemical shift assignments were then imported into PONDEROSA [21] for the automated assignment of NOE cross-peaks in ^15^N-edited NOESY and ^13^C-edited NOESY data sets. SPARKY was again used to manually validate and refine NOE peak identification and assignments. Curated lists of NOE assignments and distance and torsion angle restraints were used to further refine the structure, through manual operation of CYANA (version 3.0) [31] followed by fine-tuned structure calculation. Hydrogen bond restraints for regions with regular secondary structure (*d*_N-O_ = 2.7 to 3.5 Å; *d*_H_^N^_-O_ = 1.8 to 2.5 Å) were then added. The torsion angle constraints, generated by a TALOS+ [32] module and executed within PONDEROSA, were validated individually, by reference to SPARKY and PyMOL [33] visualizations, to remove any constraints that were too tight. Once an acceptable structure was obtained, as validated by the PSVS suite server [34], the metal-coordinating side chains were identified (C_51_, C_53_, C_111_, H_113_), and a zinc ion was added to the model. Subsequent CYANA calculations provided covalent distance restraints for the zinc coordination side chains (Cys S^γ^−Zn = 2.40 Å and His N^ε2^−Zn = 2.20 Å). The 15 best models from a total of 200 models annealed from random structures were chosen, on the basis of lowest energy with fewest violations, to represent the structure of C2 2A^pro^. With reference to the A2 (2hrv), CB4 (1z8r) and EV71 (4fvd) orthologs, MOLMOL [35] was used to superimpose the files, then calculate the root mean square deviation (rmsd) for each pair. PyMOL (version 1.2r3pre, Schrödinger, LLC) was used for graphical display. Electrostatic potential surfaces were calculated with the APBS plug-in [36] for PyMOL according to PQR files generated from Poisson-Boltzmann electrostatics calculated by the PDB2PQR package [37]. Secondary structure features in the lowest-energy model were identified by STRIDE [38]. MolProbity [39], PROCHECK [40], and the PSVS suite server [34] were used to assess the quality of the final ensemble of structures. The coordinates and related data are deposited in Protein Data Bank with the assignment code, 2M5T. The chemical shift data are deposited in the Biological Magnetic Resonance Bank, as 19079.

#### Dynamics

^1^H-^15^N NOE and ^15^N relaxation (*T*_1_, *T*_2_) data were recorded on the Agilent VNMRS 800 MHz spectrometer equipped with a conventional triple-resonance probe. Multi-interleaved NMR spectra were collected with relaxation delays of 0, 50, 100, 200, 300, 400, 600, 1200, and 1600 ms for the ^15^N *T*_1_ measurements, and with relaxation delays of 10, 30, 50, 70, 90, and 110 ms for the ^15^N *T*_2_ measurements. The relaxation rate constants were extracted in SPARKY by fitting the decay of peak height as a function of the relaxation delay to a single exponential function. Interleaved 2D ^1^H-^15^N HSQC spectra, with and without 5-s proton saturation, were collected for the ^1^H-^15^N NOE measurements. The ^1^H-^15^N heteronuclear NOE values were obtained from the ratios of peak heights between two spectra calculated with SPARKY and LibreOffice spreadsheet programs.

#### Exposure of Aromatics

The surface accessibility of aromatic side chains (His, Phe, Trp, Tyr) were evaluated for the lowest energy structure using STRIDE [38]. The observed accessible surface areas were divided by values representing the fully exposed residue accessible surface areas in corresponding tripeptides: Gly-His-Gly: (1.94 Å^2^), Gly-Phe-Gly: (2.18 Å^2^), Gly-Trp-Gly (2.59 Å^2^), and Gly-Tyr-Gly: (2.29 Å^2^) according to described procedures [41]. The residues were binned into “exposed” (30–100%), “partially exposed” (10–30%) and “buried” (0–10%) categories, accordingly. Similar procedures were used in the analysis of the three other structures: A2, CB4, EV71.

## Results

### Protein Characterization

**Figure 3:**
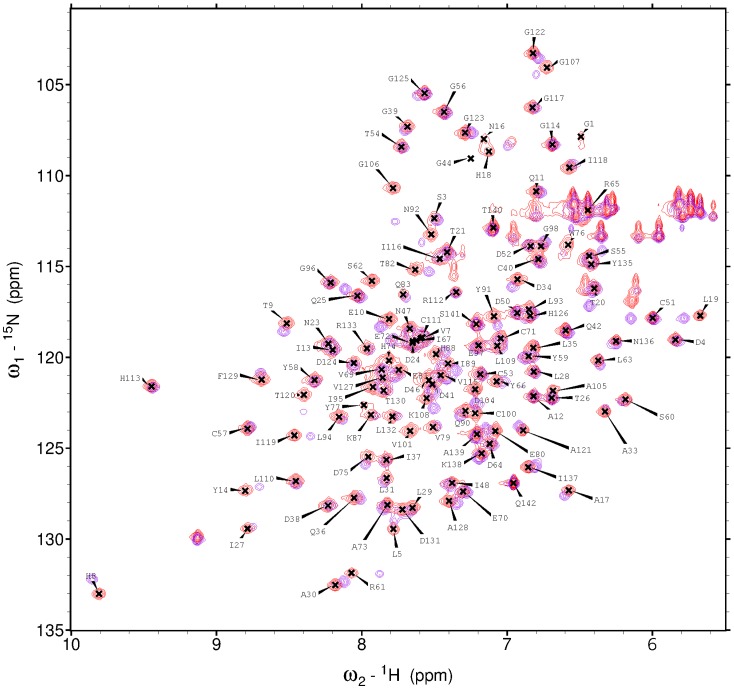
^1^H-^15^N HSQC spectra of ^15^N-labeled wild-type 2A^pro^ (*purple*) and C_105_A 2A^pro^ (*red*). The two spectra are very similar; however, that of the wild-type protease exhibits small signals attributed to self-cleavage products.

The wild-type protein was highly active [9], and the ^1^H-^15^N HSQC spectrum of ^15^N-labeled wild-type 2A^pro^ (Figure 3) was well dispersed, indicating that the protein was well folded. However, the wild-type protein aggregated over time, which prevented the collection of the valid series of three-dimensional data sets required for a structure determination. The inactive C_105_A variant, which yielded a very similar ^1^H-^15^N HSQC spectrum (Figure 3), was better behaved. Analytical gel filtration using a Shimadzu Prominence HPLC system identified conditions under which the C_105_A protein was monomeric (100 mM succinate buffer, pH 5.5, 100 mM NaCl, 2 mM TCEP), and these conditions, when evaluated by differential scanning fluorimetry (DSF), indicated that C2 2A^pro^ (C_105_A) was of sufficient stability for structure determination.

### Structure Description

**Figure 4:**
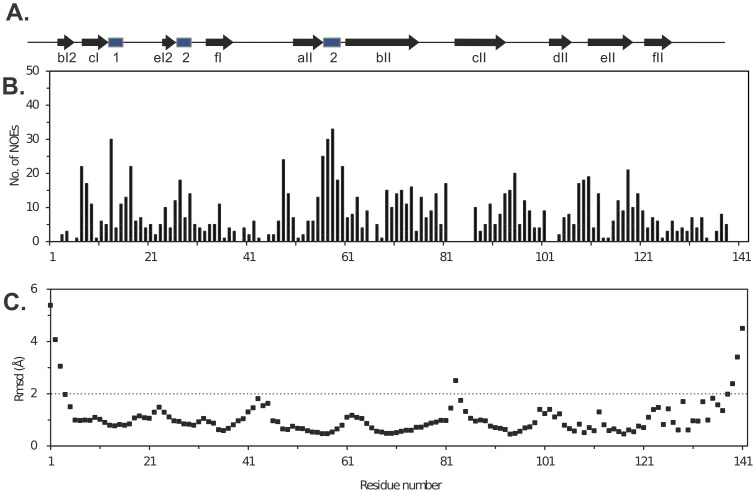
Properties of C2 2A^pro^ datasets. (A) Secondary structural features from the NMR solution structure: β-strands (*arrows*) and 3_10_ helices (*boxes*). (B) The total number of constraints used for the structure calculation plotted as a function of residue number. (C) Rmsd values for backbone atoms (N, C^α^, and C′) of the best 15 models relative to the average structure. Structurally compact regions have rmsd values below 2 Å.

The final structure was based on a total of 1440 constraints (1239 distance constraints, 142 angle constraints, and 59 hydrogen bond constraints). STRIDE [34] analysis of the structures determined that the protein consists mostly of β-strands as also reported for the ortholog, A2 2A^pro^ [11]. The assigned secondary structural elements are indicated in Figure 4A. The nomenclature follows that for A2 2A^pro^. The NOE restraints per residue used in the structure calculation are summarized in Figure 4B. The lack of NOE assignments for the N-terminus, C-terminus, and for residues 82–86 facing the catalytic triad region (H_18_, D_34_, A_105_) led to slightly higher rmsd values and lower structural compactness of the models in these regions (Figure 4C).

**Figure 5:**
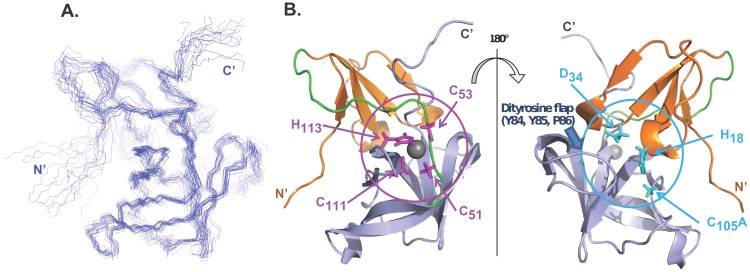
Solution structure of C2 2A^pro^. (A) The backbone atoms (N, C^α^, C′) for the best 15 models as superimposed by MOLMOL*^31^* for the regions of regular secondary structure. (B) Ribbon diagram of the lowest energy model indicating the N-terminal domain (*orange*), C-terminal domain (*gray*), and the connecting loop (*green*). Stick representations (*magenta*) show the side chains (C_51_, C_53_, C_111_, H_113_) ligating the zinc ion (*gray sphere*), and side chains of the residues (*cyan*) forming the catalytic triad (H_18_, D_34_, C_105_A). The di-tyrosine flap (Y_84_, Y_85_, P_86_) lies near this triad. The two structures are rotated by 180^o^.

The 15 best models (Figure 5A) were chosen to represent the solution structure of the full enzyme (142 amino acids). For the regions with regular secondary structure, the rmsd was 0.6 Å for backbone heavy atoms and 0.8 Å for all heavy atoms. When tested by MolProbity [39], 93.6% of the backbone angles were in “most favored” regions, 6.4% in “allowed” regions, and none in “disallowed regions” of the Ramachandran plot. The *Z*-scores for backbone/all dihedral angles from PROCHECK [40] were measured in the range of −2.95 to −5.62, while the mean score/*Z*-score values from MolProbity [39] were 24.03 to −2.60 (Table 2).

**Table 2:** Statistics for the NMR Structure of C2 2A^pro^.

Conformationally restricting distance constraints	
Intraresidue [i = j]	274
Sequential [(i–j) = 1]	181
Medium Range [1<(i–j)≤5]	148
Long Range [(i–j)>5]	636
Total	1239
Dihedral angle constraints	
φ	70
ψ	72
Hydrogen-bond constraints	59
CYANA target function [Å]	3.49
Average rmsd to the mean CYANA coordinates [Å]	
Regular secondary structure elements, backbone heavy^a^	0.6
Regular secondary structure elements, all heavy atoms^a^	0.8
Backbone heavy atoms N, Cα, C′ (1–142)	1.5
All heavy atoms (1–142)	1.7
PROCHECK Z-scores (φ and ψ/all dihedral angles)	−2.95/−5.62
MolProbity Mean score/Z-score	24.03/−2.60
Ramachandran plot summary for selected residue ranges from PROCHECK [%]^a^	
Most favored regions	85.0
Additionally allowed regions	13.2
Generously allowed regions	1.8
Disallowed regions	0.0
Ramachandran plot summary for selected residue ranges from MolProbity [%]^a^	
Most favored regions	93.6
Allowed regions	6.4
Disallowed regions	0.0
Average number of distance constraint violations per CYANA conformer	
0.2–0.5 Å	11
>0.5 Å	0
Average number of angle constraint violations per CYANA conformer	
>10°	0

a Stretches of regular secondary structure: 7–9, 12–16, 28–30, 35–39, 55–60, 65–74, 78–79, 88–96, 108–110, 115–122, 127–131.

C2 2A^pro^ has N- and C-terminal domains connected by a central loop. The N-terminal domain (Figure 5B *orange*) has four strands that constitute an antiparallel β-sheet (β-strands V_7_–T_9_ [bI2], A_12_–N_16_ [cI], L_28_–A_30_ [eI2], L_35_–G_39_ [fI]). The C-terminal domain (Figure 5B *gray*) has six strands that constitute an antiparallel β-barrel (β-strands S_55_–S_60_ [aII], R_65_–V_79_ [bII], H_88_–E_97_ [cII], G_107_–L_110_ [dII], V_115_–G_123_ [eII], H_126_–D_131_ [fII]). The connecting loop (Figure 5B *green*) includes C_40_–T_54_. The di-tyrosine flap (Y_84_, Y_85_, P_86_), conserved structurally in all such proteases, configures here as a β-hairpin loop (Figure 2C *block arrow*), as it does in A2 2A^pro^ (Y_85_, Y_86_, P_87_), CB4 2A^pro^ (Y_89_, Y_90_, P_91_), and EV71 2A^pro^ (Y_89_, Y_90_, P_91_). Three short 3_10_-helices seen in A2 2A^pro^ were also identified in the C2 2A^pro^ structure, each consisting of three residues that come after β-strands (cI, eI2, and aII); the third 3_10_-helix seen in these two proteins is missing in CB4 2A^pro^, while the second helix is categorized as an α-helix in EV71 2A^pro^.

### Protein Dynamics

**Figure 6:**
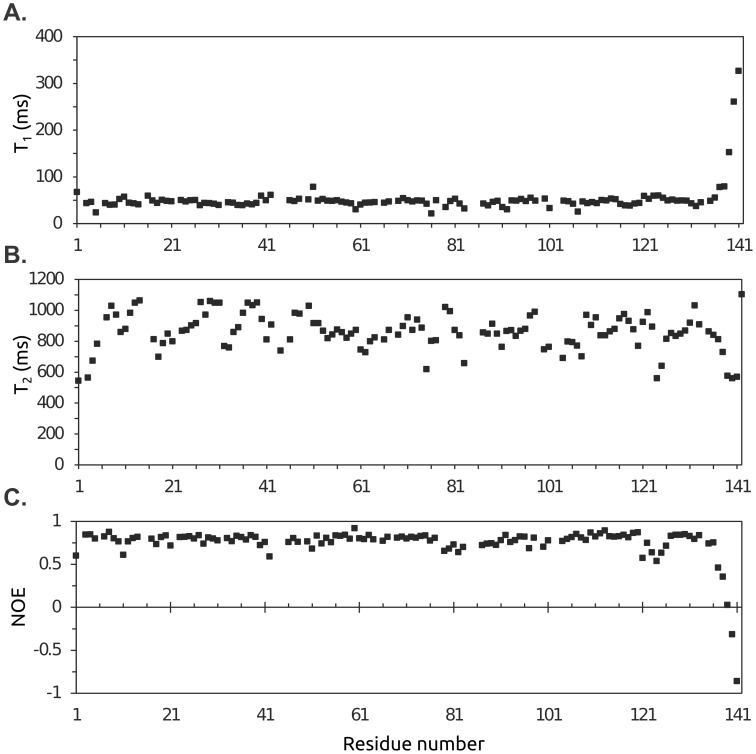
Relaxation times and heteronuclear NOEs. (A) Longitudinal (*T*_1_) relaxation times, (B) transverse (*T*_2_) relaxation times, and (C) ^1^H-^15^N heteronuclear NOE data for the nitrogen backbone atoms of C2 2A^pro^ plotted as a function of the amino acid sequence. The standard errors for all measurements were within the size of the data points shown.

Longitudinal (*T*_1_) and transverse (*T*_2_) ^15^N relaxation data as well as ^1^H-^15^N heteronuclear NOE data (Figure 6) were collected to explore the dynamic behavior of C2 2A^pro^. We used Eq. 1 to estimate the overall correlation time (*τ*_c_) from the *T*_1_/*T*_2_ ratios of residues involved in elements of secondary structure.

 (1)

The resulting *τ*_c_ value was 10.5 ns. Inspection of the *T*_1_/*T*_2_ ratios and ^1^H-^15^N heteronuclear NOE data showed, apart from the five mobile C-terminal residues, very little internal motion over the whole sequence, including the loop regions. This appears to be a common feature of picornaviral proteases [12]. However, despite little evidence for internal motion, the non-uniform intensity of peaks in ^1^H-^15^N -HSQC spectra suggests the existence of localized structural heterogeneity. CB4 2A^pro^ exhibited similar phenomena in previous NMR studies [14].

## Discussion

### NMR Methods

The methods used in this study represent a collaborative effort by CESG and NMRFAM to develop generalized, rapid-through-put techniques for protein purification and structure determination. This charged, self-cleaving protease with a tendency to aggregate presented particular challenges. The problems were solved here, by stepwise judicious selection of cloning vector (pE-SUMO), host strain, isolation and purification protocols, the C_105_A mutation, and solution conditions. Linkage of the output from PINE-NMR [30] to PINE-SPARKY validations [20] facilitated and virtually automated the spectral peak assignments. The final structure was of high quality and well supported by the extensive datasets.

### 2A^pro^ Structure Comparisons

**Figure 7:**
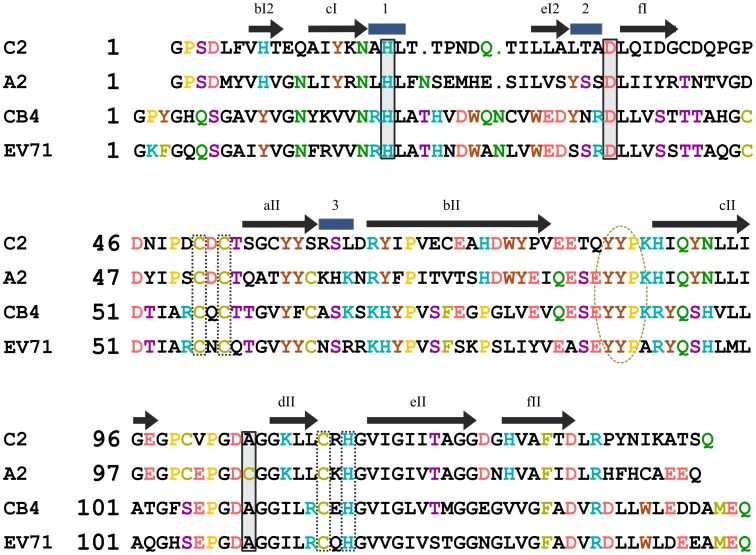
Sequence alignment of C2, A2, CB4, and EV71 2A^pro^. Residues are color-coded by type. Residues in the catalytic triad (C2: H_18_, D_34_, and C_105_A) are boxed with solid lines. Residues whose side chains ligate the zinc ion (C2: C_51_, C_53_, C_111_, H_113_) are boxed with dashed lines. The ellipse highlights the conserved YYP sequence in the di-tyrosine flap. Symbols above the sequences indicate secondary structural features as per Figure 3.

The C2 2A^pro^ is the first protein from an RV-C to be examined at the structural level. Among enteroviruses, the only viral genus to have such enzymes, structures were previously reported for 2A^pro^ from RV-A2 [11] and EV-71 [13] determined by crystallography and EV-CB4 [14] determined by NMR. The sequence identities are 57% between A2 and C2, 41% between CB4 and C2, and 40% between EV71 and C2. Structure alignments show that the only relative indels are confined to a short stretch in the first domain (before eI2) and to length discontinuities at the N- and C-terminal cleavage sites (Figure 7). For comparison, important structural and functional elements are highlighted on this map. The substrate-binding di-tyrosine flap (YYP) is marked by an ellipse. The one His (H_113_) and three Cys residues (C_51_, C_53_, C_111_ *dashed boxes*) responsible for coordinating the structural zinc ion (Figure 5B *gray sphere*) converge on the back side of the molecule, basically holding the main domains together. Sequencing studies have highlighted a number of RV isolates that are apparent recombinants within the 2A^pro^ region [42]. When this occurs, invariably, within or between RV-A and RV-C strains, the identified breakpoints cluster in the central linker region and at the C-terminus, swapping the intact N- and C-terminal domains. That these recombinants are apparently fully functional suggests that the two main domains fold independently, with each domain contributing zinc coordination elements that stabilize the full enzyme.

The catalytic triads (H_18_, D_34_, C_105_) in all four structurally determined enzymes are identical (Figure 7 *solid boxes*) and located within a pronounced substrate-binding groove opposite to the zinc. The C_105_ nucleophile is in a conserved PGDCGG motif, between two β-strands within the C-terminal domain (cII and dII). In the C2, as well as the CB4 and EV71 structures, this reactive Cys was mutated to Ala to obtain protein sufficiently stable for structure determination. The sequences indicated (Figure 7) reflect those mutations.

**Figure 8:**
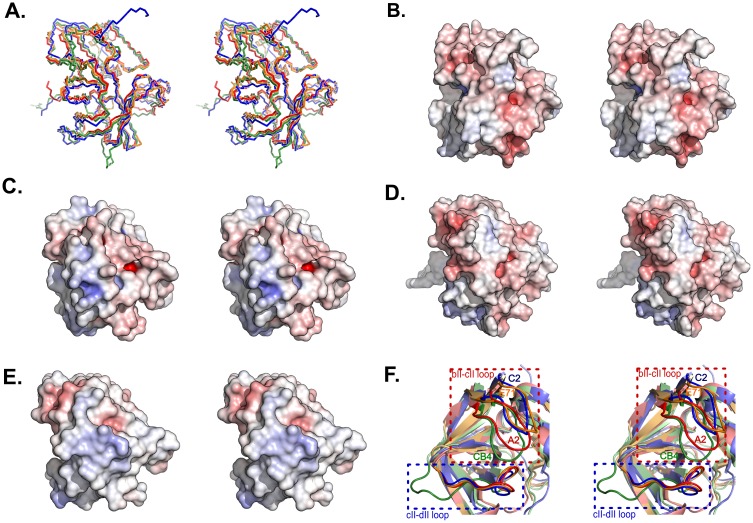
Cross-eyed stereoscopic representations of 2A^pro^ structures. (A) Superimposition of backbones of the four proteases showing their structural similarity. Pairwise rmsd values for C2 relative to both A2 and CB4 proteases are both 1.809 Å, while to EV71 protease is 1.4 Å. Poisson-Boltzmann electrostatic potential surfaces are illustrated by PyMOL [29] for (B) C2, (C) A2,(D) CB4 and (E) EV71 2A^pro^. Each structure is shown in the same orientation. (F) Comparison of the positions of the bll−cll and cll−dll loops in the structures of C2 (*blue*), A2 (*red*), CB4 (*green*), and EV71 (*orange*) 2A^pro^.

Superimposition of the 3D structures of C2 and CB4 2A^pro^ (Figure 8A; NMR model 1) gave a lower pairwise backbone rmsd (1.809 Å) than might have been expected from the 41% sequence identity. Superimposition of C2 and EV71 2A^pro^ models (40% sequence identity) yielded the lowest pairwise rmsd (1.4 Å). When electrostatic potential surfaces were generated with the contouring value set to ±10 kT/e (Figure 8 B,C,D,E), all four enzymes exhibited similar negative charge surface distributions (*red*) despite the overall sequence differences. However, the C2 enzyme (Figure 8B) lacks several intensely basic surface patches (*blue*) displayed by A2 (Figure 8C), CB4 (Figure 8D) and EV71 (Figure 8E). Examples of sequence differences at aligned positions that result in a more acidic p*I* for the C2 sequence overall (4.62) than for A2 (5.43), CB4 (5.20), or EV71 (6.04) include C2 G_39_/A2 R_40_ and C2 L_63_/A2 K_64_. Actually, the C2 enzyme has the most acidic p*I* of known 2A^pro^ sequences [8], [9].

Other differences between the four structures are observed in the distance between the two loops (bII-cII and cII-dII) that constitute the binding cleft (Figure 8F). The two loops are closest together in the structure of CB4 2A^pro^ (*green*) followed by A2 2A^pro^ (*red*), and the binding sites of these two proteases can be characterized as closed. By contrast, EV71 2A^pro^ (*orange*) and C2 2A^pro^ (*blue*) exhibit open binding sites with their two loops about the same distance apart.

Instead of positive charges, the C2 2A^pro^ structure exposes an unusual level of aromatics on its surface. In most other proteins, aromatics normally contribute to the hydrophobic core that stabilizes the protein structure [43]. The degree of exposure for each residue of C2 2A^pro^ was determined by comparing the observed solvent accessible surface area (SAS), obtained from STRIDE [38], to theoretical SAS values for a fully exposed residue. By this metric, 12 of 18 (67%) aromatic residues in C2 2A^pro^ were found to be exposed to solvent (6 Tyr, 4 His, 1 Phe, 1 Trp). Four more are only partially buried (2 Tyr, 2 His), and only two are fully (>90%) buried (Y_58_, F_129_). Similar analysis of the other structures showed the exposure of 12 of 26 (46%) aromatics in A2 2A^pro^ (5 Tyr, 6 His, 1 Trp), 12 of 22 (55%) aromatics in CB4 2A^pro^ (4 Tyr, 5 His, 1 Phe, 2 Trp), and 11 of 20 (55%) aromatics in EV71 2A^pro^ (5 Tyr, 4 His, 2 Trp). Rather than aromatics, the hydrophobic core of C2 2A^pro^ consists mostly of Val, Leu and Ile residues, an unusual selection for this purpose. Similar characteristics were noted for CB4 2A^pro^ [14]. Of the four proteins, C2 2A^pro^ has the highest ratio of exposed aromatics and also the surface with the lowest positive charge.

### RV 2A^pro^ Sequence and Structural Variability

Comparison of the four structures now available supports the idea that the hallmark sequence variability among enterovirus 2A^pro^ translates mostly into surface charge variability, rather than alterations in the essential core configuration, the loop lengths, or internal dynamics that might affect the catalytic residues [14]. These are relatively rigid proteases, and yet in infected cells, different RV isolates are quite selective about their substrate preferences and rates of cleavage [7], [17]. To date, the preferences of only six RV enzymes (A16, A89, B4, B14, C2, C6) have been compared head-to-head [9], although seven more (A1, A2, A45, A95, B17, B52, C15) were recently cloned and are undergoing similar tests (K. Watters and A. C. Palmenberg, unpublished). Polyclonal antibodies raised against the A16 enzyme cross-react with C15 but not C2 (Watters and Palmenberg, 2011), verifying differences at the surface level, but also suggesting the general 2A^pro^ proclivities may eventually cluster into a limited series of reactive clades, along sequence (e.g. A16 and C15) or species (A or B or C) lines. Because many of the preferred, natural Nup substrates for 2A^pro^ lie buried in the hydrophobic cores of the nuclear pores, perhaps the surface groupings influence physical accessibility, contributing at least in part to the observed cleavage patterns. Surface differences between the A2 and CB4 enzymes have been shown to directly affect the relative rates of eIF4G cleavage [44].

**Figure 9:**
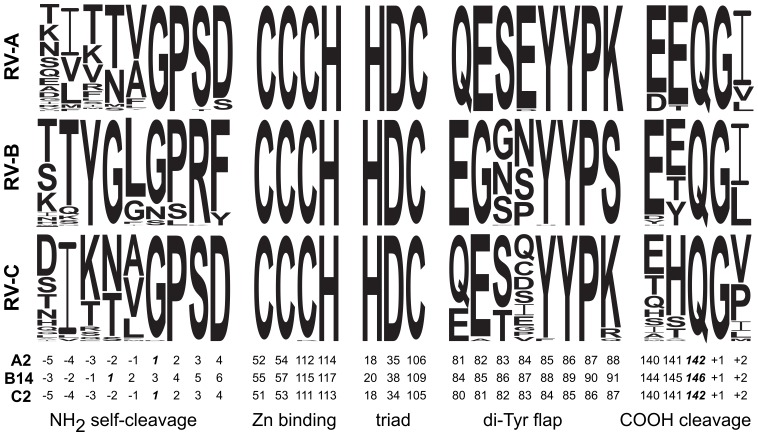
RV sequences by species. WebLogo depictions [46] summarize full species alignment information for key 2A^pro^ residues. RV polyprotein alignments have been described [8]. This dataset compared RV-A (79 types, 208 seqs), RV-B (30 types, 74 seqs), RV-C (32 types, 67 seqs). The residue height indicates the relative amino acid frequency. The A2, B14 and C2 numbering system is for the native, ungapped proteins.

Another possibility is that the substrate binding pocket, sensitive to the P8−P2′ sequence of the substrate, is the key to specificity [15]. Created in part by the variable di-tyrosine flap, the binding groove is responsive, even during the autocatalytic self-cleaving event, to the sequence and shape of the substrate that fills it. When nine amino acids flanking the NH_2_-terminus of B14 2A^pro^ were substituted into an A1 or A2 context, the chimeras were unable to cleave themselves from their polyproteins [45]. The same was true when the A2 enzyme was tested in *trans* against peptides encoding other RV processing sites, even those from closely related viruses [16]. It required at least three substitutions within this length to re-establish activity. The protease reacted to mutated residues in the P2, P1 and P2′ locations during *cis* reactions [45], but is apparently tolerant of certain changes in the P1, P2′, and P3′ locations during *trans* reactions [16]. Clearly, all these enzymes are sensing both the shape and sequence of their targets [14]. A WebLogo depiction [46] summarizing all known RV sequences within the self-cleavage sites (Figure 9) highlights the variability encoded here. Not only are the RV-B enzymes extended by two amino acids (cleavage is between positions “−1” and “1”), there is almost no consensus within or between species. The di-tyrosine flap, both upstream and downstream of the few conserved residues (YYP) is another region with pronounced variability. The flap forms one side of the binding cleft (Figure 5B) where substrate acceptance is a prerequisite to the conformational changes that occur during catalysis. In contrast, the zinc-binding residues, the catalytic triad, and C-terminal di-peptide (Q/G) recognized by 3C^pro^ are absolutely conserved in all species, types, and isolates (n = 348). The 3C^pro^ enzymes as a rule have more limited selectivity, and for all RV, the carboxyl terminus of 2A^pro^ is released at an identical Gln/Gly pair.

The current determination of the structure of C2 2A^pro^ is only the start of further investigations that compare and contrast this important cohort of enzymes. It has been proposed that the particular avidities with which individual 2A^pro^ attack their Nups (or eIF4G) profoundly affect relative viral replication levels, intracellular signaling or extra cellular signaling, all of which are underlying triggers for different host immune responses [9]. It is important to define these mechanisms, embedded in the structures, in order to understand the consequent variability among virus phenotypes.

## Associated Content

### Accession Codes

The atomic coordinates and assigned chemical shifts and structural constraints were deposited in the PDB with ID code 2M5T. NMR data were deposited in the BMRB with ID code 19079.
